# Drug Discovery of DKK1 Inhibitors

**DOI:** 10.3389/fphar.2022.847387

**Published:** 2022-03-09

**Authors:** Hewen Jiang, Zongkang Zhang, Yuanyuan Yu, Hang Yin Chu, Sifan Yu, Shanshan Yao, Ge Zhang, Bao-Ting Zhang

**Affiliations:** ^1^ School of Chinese Medicine, Chinese University of Hong Kong, Hong Kong, China; ^2^ Guangdong-Hong Kong Macao Greater Bay Area International Research Platform for Aptamer-Based Translational Medicine and Drug Discovery, Hong Kong, China; ^3^ Law Sau Fai Institute for Advancing Translational Medicine in Bone and Joint Diseases, School of Chinese Medicine, Hong Kong Baptist University, Hong Kong, China; ^4^ Institute of Integrated Bioinformedicine and Translational Science, School of Chinese Medicine, Hong Kong Baptist University, Hong Kong, China

**Keywords:** drug discovery, wnt signaling, DKK1 inhibitors, antibodies, small molecule inhibitors, nucleic acid inhibitors

## Abstract

Dickkopf-1 (DKK1) is a well-characterized Wnt inhibitor and component of the Wnt/β-catenin signaling pathway, whose dysregulation is associated with multiple abnormal pathologies including osteoporosis, Alzheimer’s disease, diabetes, and various cancers. The Wnt signaling pathway has fundamental roles in cell fate determination, cell proliferation, and survival; thus, its mis-regulation can lead to disease. Although DKK1 is involved in other signaling pathways, including the β-catenin-independent Wnt pathway and the DKK1/CKAP4 pathway, the inhibition of DKK1 to propagate Wnt/β-catenin signals has been validated as an effective way to treat related diseases. In fact, strategies for developing DKK1 inhibitors have produced encouraging clinical results in different pathological models, and many publications provide detailed information about these inhibitors, which include small molecules, antibodies, and nucleic acids, and may function at the protein or mRNA level. However, no systematic review has yet provided an overview of the various aspects of their development and prospects. Therefore, we review the DKK1 inhibitors currently available or under study and provide an outlook on future studies involving DKK1 and drug discovery.

## 1 Introduction

Wnt signaling has been the focus of drug discovery and immunotherapy since it was discovered to be involved in developmental processes during embryogenesis, including cardiovascular development and neural development, as well as a variety of cell behaviors such as cell proliferation, migration, adhesion, and polarity (MacDonald et al., 2009). As the first Wnt pathway to be identified, the canonical Wnt signaling transduction pathway has been extensively investigated (Moon et al., 2002; Logan and Nusse, 2004; Westendorf et al., 2004; Chen et al., 2008; Zhang et al., 2015b). Despite the success of modern medicine in treating various diseases, many patients still suffer from illnesses related to dysfunction of canonical Wnt signaling. The Wnt pathway is mediated by Wnt proteins, which are pivotal cysteine-rich glycoproteins secreted to modulate specific downstream target genes. Over the past 40 years, many studies have highlighted the importance of developing drugs to stimulate the Wnt pathway and exploring possible therapeutic strategies. Given the prohibitive cost of obtaining biologically active Wnt proteins for use in development of agonists, a feasible alternative approach to activating the Wnt pathway is to block naturally existing inhibitors (Hoeppner et al., 2009). On the other hand, it is well known that DKK1 functions as an endogenous suppressor of the canonical Wnt signaling pathway; it also participates in other signaling pathways, including the c-Jun NH_2_-terminal kinase (JNK) pathway and the DKK1/CKAP4 pathway. High expression of DKK1 has been detected in various diseases, and DKK1-mediated repression has been confirmed to be the cause of numerous abnormalities (Niida et al., 2004; Mazon et al., 2016). Owing to the significance of DKK1 in Wnt signaling, effective strategies to treat diseases related to this signaling pathway could include developing potently efficient inhibitors such as antibodies and small molecules to target the function of DKK1 or suppressing DKK1 expression at the protein and gene levels. Many studies of DKK1 inhibitors have been carried out in recent years, and several anti-DKK1 antibodies have been developed and tested in clinical trials (Zhou et al., 2013; Kimura et al., 2016). However, systematic reviews of these inhibitors are lacking. In this review, we summarize the reported inhibitors of DKK1 with the aim of helping researchers to gain a comprehensive understanding of current developments and future perspectives regarding DKK1 inhibitors.

## 2 Structure and Biological Function of Dickkopf-1

### 2.1 Source, Structure, and Function of Dickkopf-1

The *dickkopf* family encodes four main secreted glycoproteins of 255–350 amino acids: DKK1, DKK2, DKK3, and DKK4 (Glinka et al., 1998; Fedi et al., 1999; Krupnik et al., 1999). DKK1, DKK2, and DKK4 have high similarity in primary sequence; however, DKK3 does not, which might be why it cannot regulate Wnt signaling by binding to the same receptors (Fedders et al., 2004). In particular, DKK1 shares up to 70% identity with DKK2 in the C-terminal region. Although DKK1/2/4 have mainly been reported to have inhibitory effects on Wnt signaling, DKK2 was also discovered to show the opposite effect, depending on the cellular micro-environment (Wu et al., 2000). DKK1 is a protein of 266 amino acids composed of two conservative cysteine-rich domains that are separated by a sequence possessing unknown functions and variable length. First discovered in *Xenopus*, it was identified as a prominent regulator involved in the induction of head formation in amphibian embryos (Fedi et al., 1999). Antibody-mediated inhibition of DKK1 confirmed that it was required for head formation (Glinka et al., 1998). DKK1 has potential N-linked glycosylation at Asn225 and two O-linked glycans attached at Ser30, resulting in a difference between its apparent molecular size and theoretical molecular weight (Fedi et al., 1999; Haniu et al., 2011). The C-terminal domain of DKK1 (DKK1c) has been identified as a necessary and sufficient domain for Wnt pathway inhibition, as well as being responsible for binding to LRP6. Conversely, the N-terminal domain does not exhibit the same function as DKK1c and even masks the ability of DKK1c to interact with LRP6 (Brott and Sokol, 2002). The function of the N-terminal domain may be associated with enhanced invasive activity and an anti-apoptotic signaling pathway that could be neutralized by the anti-DKK1 antibody. As of 2011, the DKK1c-LRP6 complex structure has been determined. DKK1 mainly binds to the third and fourth propeller domains of LRP5/6, and the loop of DKK1c (residues 222–231) is the main binding site. Other interactions including a salt bridge between LRP6 Asp830 and DKK1 Arg191 also exist (Cheng et al., 2011). However, the structure of full-length DKK1 and its complex with LRP6 remains unknown.

### 2.2 Signaling Pathways Related to Dickkopf-1

#### 2.2.1 Wnt-β-Catenin Signaling Pathway

**FIGURE 1:**
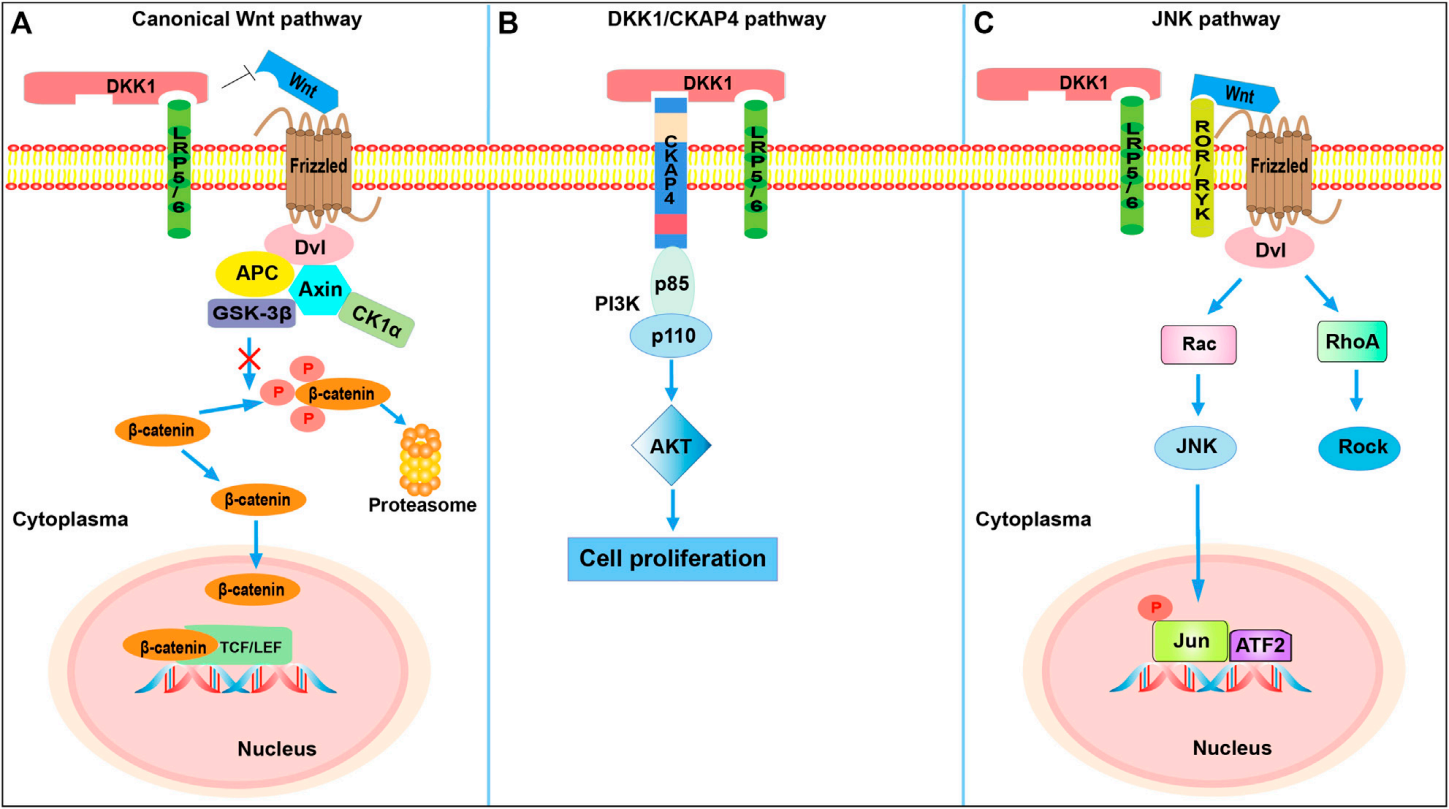
The DKK1-involved signaling pathways. **(A)** DKK1-mediated inhibition of canonical Wnt signaling. DKK1 inhibits β-catenin-dependent Wnt signaling by binding to the LRP5/6 co-receptor and blocking Wnt binding, which results in β-catenin degradation. **(B)** DKK1-mediated activation of PI3K/Akt signaling. Binding of DKK1 to the CKAP4 receptor activates PI3K/Akt signaling and stimulates cell proliferation. When DKK1 binds to both LRP6 and CKAP4, cell proliferation is further promoted compared with binding of CKAP4 alone. **(C)** DKK1 activation of JNK pathway. The competitive binding of DKK1 to LRP5/6 shifts Fz receptors to the JNK pathway. Dvl, Dishevelled; Axin, axis inhibition protein; APC, adenomatous polyposis coli; GSK-3β, glycogen synthase kinase 3β; CK1α, casein kinase 1α; TCF, T-cell factor; LEF, lymphocyte-enhancer-binding factor; CKAP4, cytoskeleton associated protein 4; PI3K, phosphoinositide 3-kinase (consisted of p85 and p110 subunits); AKT, protein kinase B; ROR/RYK, receptor-like tyrosine kinase; Rac, Rac family Small GTPase; JNK, Jun N-terminal kinase; ATF2, activating transcription factor 2; RhoA, RAS homolog gene-family member A; Rock, Rho-associated coiled-coil-containing protein kinase.

Wnts belong to the glycoprotein family. This family has 19 members, which are typically secreted glycoproteins of 350–400 amino acids that exert pivotal functions in stem cell maintenance and development *via* the Wnt-β-catenin signaling pathway (Nusse, 2005). This canonical Wnt signaling pathway has been extensively investigated and is involved in many stages of invertebrate and vertebrate development and adult tissue homeostasis (Wodarz and Nusse, 1998; Logan and Nusse, 2004). Mechanistic studies have revealed that the transduction of Wnt-β-catenin signaling mediated by Wnts is activated through interplay with seven-pass transmembrane receptors of the Frizzled (Fz) family and LRP5/6 (Wodarz and Nusse, 1998). Upon binding of Wnts to the conservative cysteine-rich domain of Fz receptors and LRP5/6, a cytoplasmic protein called Dishevelled (Dvl) is stimulated. Dvl can block axis inhibition protein (Axin), preventing formation of the destruction complex consisting of adenomatous polyposis coli, Axin, glycogen synthase kinase-3β, and casein kinase-1α. This in turn prevents the phosphorylation and consequential degradation of β-catenin, a key tumor cell proliferation regulator within the Wnt-β-catenin signaling pathway (Figure 1A). Subsequently, β-catenin is stably translocated into the nucleus and binds to the N-terminus of lymphoid enhancer factor/T cell factor transcription factors, activating transcription of the downstream target genes and thus exerting the corresponding functions (Behrens et al., 1996; Niida et al., 2004; Chamorro et al., 2014). However, several natural extracellular antagonists, including the Fz-related protein sFRP, Wnt inhibitory factor 1, sclerostin, Wise, and DKK1, exert inhibitory effects through competitively binding LRP5/6 to effectively block associations between Wnts and LRP5/6, thereby attenuating the expression of downstream genes in the canonical Wnt signaling pathway (Piccolo et al., 1999; Itasaki et al., 2003). Among them, DKK1, a typical Wnt antagonist, antagonizes Wnt signaling by triggering the internalization of coreceptor LRP5/6 (Semënov et al., 2001). DKK1 has also been found to participate in the Wnt pathway by binding to Kremen, a transmembrane protein interacting with DKK1 with high affinity; however, Kremen might not be a necessary component in DKK1-mediated Wnt antagonism (Mao et al., 2002; Wang et al., 2008). Overall, a wide range of functions of the Wnt pathway influencing developmental decisions in humans or animals have been uncovered over the years. However, gaining further insight into several outstanding mechanisms of signaling transduction and developing interventions for the treatment of diseases remain formidable challenges (Logan and Nusse, 2004).

#### 2.2.2 c-Jun NH_2_-Terminal Kinase Pathway

The JNKs, also called stress-activated protein kinases, form a well-identified subfamily of mitogen-activated protein kinases (Kyriakis and Avruch, 2001; Weston and Davis, 2002). They were initially found to catalyze the phosphorylation of the N-terminal transactivation domain of c-Jun (Ser-63 and Ser-73), leading to the inhibition of its ubiquitination, which was the origin of the name “JNK.” However, JNK can also phosphorylate other transcription factors, including JunB, JunD, c-Fos, and ATF, all of which have critical roles in controlling the levels of several stress-responsive genes (Yang et al., 1998). There are three main mammalian JNK genes, JNK1, JNK2, and JNK3, located on different chromosomes. Their expression and functions vary among individual cells or organs, owing to differences in substrate affinity between the multiple JNK isoforms resulting from splicing of JNK genes (Dérijard et al., 1994). The JNK pathway is involved in many neurological disorders (Savage et al., 2002), diabetes (Davis et al., 2000; Hirosumi et al., 2002), inflammatory bowel disease (Malamut et al., 2006; Craige et al., 2019), non-alcoholic fatty liver (Ferreira et al., 2011), heart diseases (Cook et al., 1999), and cancers (Hui et al., 2008; Chang et al., 2009; Barbarulo et al., 2013). Previous studies have also demonstrated that JNK functions are important in apoptosis in a variety of cell death types, including necrosis and autophagy, and that the loss of JNK signaling can cause suppressed tumor necrosis factor (TNF)-stimulated cell death (Ventura et al., 2004).

The expression of DKK1 has been reported to be dependent on the Ap-1 family member c-Jun. DKK1 levels and bone morphogenetic protein (Bmp)-triggered apoptosis were found to be considerably reduced in c-Jun^−/−^ mice, indicating a pivotal role of c-Jun in transcriptional activation of DKK1 and regulation of Bmp signaling (Grotewold and Rüther, 2002). In addition, the apoptosis induced by Bmp requires c-Jun to activate DKK1, potently inhibiting the propagation of Wnt signals. Hence, the Bmp signaling pathway is likewise entangled with DKK1. Some evidence suggests that JNK signaling occurs *via* the β-catenin-independent Wnt pathway. In prostate cancer cells, ovarian cancer cells, and osteosarcoma, JNK signaling was activated by DKK1; in these cases, β-catenin levels were not affected, and promotion of cell invasion or tumor growth was observed. One proposed hypothesis for the activation of JNK signaling is that competitive binding of DKK1 to LRP5/6 could shift the Fz receptors to the JNK pathway because of the higher availability of Wnts (Kagey and He, 2017). When Wnts bind to coreceptors ROR and RYK, the recruited Dvl contributes to the activation of Rac GTPase, which activates JNK (Figure 1B). However, more evidence is needed to elucidate the mechanism more precisely. Dvl-dependent RhoA signaling is also activated, and this cascade promotes actin cytoskeletal reorganization and cell movement (Weston and Davis, 2007). Colla et al. (2007) reported that the activation of JNK signaling induced DKK1 expression in multiple myeloma (MM; a neoplastic disorder of plasma cells in the bone marrow) plasma cells. In their study, DKK1 was found to be responsive to the JNK signaling cascade, and they assumed that the sensitivity of myeloma cells in the early stages to various stresses activated JNK signaling, thereby increasing DKK1 expression.

#### 2.2.3 Dickkopf-1/Cytoskeleton-Associated Protein 4 Pathway

Cytoskeleton-associated protein 4 (CKAP4), also termed p63, ERGIC-63, or CLIMP-63, is a type-II transmembrane protein of 63 kDa mainly localized to the endoplasmic reticulum (Bhavanasi et al., 2016). DKK1 binds to receptor LRP5/6 through a cysteine-rich domain (CRD1; DKK1c); CKAP4 has recently been identified as another DKK1 receptor, where the interactive domain is CRD2 (the N domain). The extracellular domain of CKAP4 directly binds to DKK1, and the K_D_ value of DKK1 towards CKAP4 is 0.42 nM. The binding affinity of DKK1-LRP6 (K_D_, about 0.34–0.5 nM) is similar to that of DKK1-CKAP4, suggesting a certain significance of CKAP4 in DKK1-related regulation (Figure 1C). Once DKK1 interacts with CKAP4, the subsequent formation of the CKAP4-PI3K (phosphatidylinositide 3-kinase) complex via the proline-rich domain of CKAP4 and the Src homology 3 domain of the p85 subunit of PI3K (composed of p85 and p110 subunits) accelerates the generation of phosphatidylinositol-3,4,5-triphosphate and thus activates AKT and stimulates cell proliferation (Kimura et al., 2016). Intriguingly, the presence of LRP6 does not compete with CKAP4 for binding with DKK1 owing to the different locations of the binding domain; instead, it enhances DKK1-CKAP4 signaling, thereby promoting cancer cell proliferation more than DKK1-CKAP4 interaction alone (Kikuchi et al., 2021). The specific mechanism of the stimulation of PI3K mediated by DKK1/CKAP4 signaling is still under investigation. Clinically, DKK1- and CKAP4-positive patients with pancreatic ductal adenocarcinoma have shorter 5-years survival and relapse-free survival than DKK1- or CKAP4-negative patients. Conversely, high expression of CKAP4 was found to be consistent with longer overall survival (OS) in patients with cholangiocarcinoma and hepatocellular carcinoma (HCC), indicating that the cellular context might influence the function of CKAP4 (Kimura et al., 2019). Furthermore, DKK1 and CKAP4 were found in tumor lesions in more than 70% of lung squamous cell carcinoma patients. However, CKAP4 expression was reduced to a nearly undetectable level in noncancerous regions. The pharmacological treatment with an anti-CKAP4 antibody suppressed tumor formation in mice, indicating the potential of CKAP4 as an anti-tumor target (Kimura et al., 2016). Surprisingly, DKK1 functions through the DKK1/CKAP4/PI3K pathway to augment the expression of plasmalemma vesicle-associated protein in cholangiocarcinoma cells, which is positively linked to angiogenesis in various tumors, hence also making this pathway a prospective anti-angiogenic target (Wang et al., 2021).

## 3 Correlative Diseases of Dickkopf-1

### 3.1 Cancers

DKK1, as a critical component in the canonical Wnt pathway, is linked to a variety of cancers (Kaiser et al., 2008; Huang et al., 2018). It has been strongly suggested that DKK1 may have a proapoptotic effect on tumor cells *via* the downregulation of β-catenin (Lee et al., 2004). In a variety of tumors, a high expression level of DKK1 is a prospective biomarker for diagnosis and prognosis. A study of tumor tissue samples from 205 lung cancer patients showed that DKK1 expression was positively linked with vasculogenic mimicry, which led to greater invasiveness of cancer cells (Yao et al., 2016). This was further confirmed by the observation of much more severe bone metastasis in 470 non-small-cell lung cancer (NSCLC) patients with high serum DKK1 levels relative to control groups (Qiao et al., 2017). Likewise, high serum DKK1 levels make DKK1 a promising biomarker in esophageal squamous cell carcinoma, gastric cancer, pancreatic cancer, prostate cancer, cholangiocarcinoma, laryngeal squamous cell carcinoma, and HCC (Shi et al., 2013; Begenik et al., 2014; Rachner et al., 2014; Shi et al., 2014; Han et al., 2015; Liu et al., 2016a; Hong et al., 2018). Elevated DKK1 expression was associated with poor prognosis in HCC patients, whereas it was independent of OS and relapse-free survival (Li et al., 2018). On the contrary, DKK1 expression was attenuated in colorectal cancer (CRC), and it downregulated expression of VEGF (a regulator of angiogenesis). Accordingly, DKK1 may be involved in repression of tumorigenesis and angiogenesis in CRC (Liu et al., 2015). Despite the low secretion of DKK1, tumor growth of MDA-MB435 melanoma cells was suppressed through the activation of cell death; this also reflects the inhibitory role of DKK1 in tumorigenicity (Mikheev et al., 2007). Such differences in the functions of DKK1 in various cancers might be caused by variations in the interplay of existing signaling pathways in varied cellular circumstances; however, further evidence is required to fully explain them.

In addition to the abovementioned cancers, DKK1 is involved in gynecological cancers. DKK1 levels are higher in cervical cancer patients, resulting in lymphatic metastasis, and are related to tumor diameter. Thus, DKK1 was identified as a good clinical and prognostic factor (Jiang et al., 2013). In ovarian cancer, the deadliest of the gynecological tumors, DKK1 overexpression provides a tumor-friendly microenvironment to inhibit the anti-tumor immune cell populations, which may have a leading role in combination immunoregulatory therapy (Bray et al., 2018; Betella et al., 2020). Serum DKK1 expression levels in 89 breast cancer patients were found to be significantly higher than those in the healthy control group, and the discrepancy was even more evident in patients with bone metastasis. A novel molecule known as dorsomorphin has been shown to significantly reduce both mRNA and protein levels of DKK1 in breast cancer cells, suggesting that it could be an effective treatment for breast cancer (Li et al., 2015; Kasoha et al., 2018). Data from 94 bladder cancer patients revealed a similar positive relationship between elevated serum DKK1 and lymph node metastasis, as well as a lower survival rate (Kaba et al., 2014; Sun et al., 2015). However, it was suggested that DKK1 secreted by perichondrium mesenchymal stem cells inhibited the growth of breast cancer, whereas its absence significantly reduced the inhibitory effects of mesenchymal stem cells on tumor cells (Li et al., 2016). Collectively, DKK1 is an important regulator in tumors and deserves more attention as a key therapeutic target.

### 3.2 Bone Diseases

Bone cells include osteoclasts, osteoblasts, and osteocytes. Their dysfunction leads to abnormal bone resorption or bone formation, which may contribute to metabolic bone disease (Gerstenfeld and Einhorn, 2006). Both preclinical and clinical data were suggestive that high DKK1 expression can impair osteoblast activity and cause bone loss (Zhang and Drake, 2012). In MM cells, it has been well established that DKK1 blocks osteoblast differentiation and thereby impedes bone formation (Gavriatopoulou et al., 2009). Levels of DKK1 in plasma cells were shown to be correlated with the presence of lytic lesions in myeloma patients; these lesions can evolve into pathological fractures. A further study of 171 patients diagnosed with MM suggested that the extent of osteolytic bone disease was positively related to the overexpression of DKK1, and forced overexpression of DKK1 in plasma cell myeloma has been shown to cause osteopenia, hematopoietic stem cell function deficits, and fracture repair inhibition (Tian et al., 2003; Politou et al., 2006; Kaiser et al., 2008; Zhou et al., 2013). Fracture repair is promoted by DKK1 blockage, and a decrease in DKK1 gene expression leads to an increase in bone mass and strength (Komatsu et al., 2010). Similarly, lumbar spine bone mineral density (BMD) is positively correlated with serum DKK1, whereas no significant association in bone metabolism, such as a link with morphometric vertebral fractures, has been discovered (Garcia-Martin et al., 2014). A common skeletal disease characterized by low BMD and impaired microarchitecture, osteoporosis (especially postmenopausal osteoporosis) is closely associated with DKK1 expression. Anti-DKK1 antibody treatment of OVX rhesus was shown to be capable of significantly increasing bone formation and BMD (Rachner et al., 2011). Notably, even in the case of implanted bones and murine femurs in nonmyelomatous SCID-rab mice treated with DKK1 antibody, a significant increase in the BMD of the nonmyelomatous implanted bone was observed (Yaccoby et al., 2007). Therefore, treatment with DKK1 antibody could be an effective means of increasing bone formation in bone loss diseases. In addition, DKK1 blockade prevented bone erosion in the sacroiliac joints and enhanced sacroiliac ankylosis, underlining the great importance of the Wnt pathway in axial joint disease (Uderhardt et al., 2010). Sacroiliac erosion and fusion have typically been characterized in ankylosing spondylitis (AS) patients, and studies published before 2012 reported higher DKK1 expression; however, contradictory results have since emerged, and a comprehensive meta-analysis found no association of serum DKK1 with AS (Wu et al., 2018). We will not elaborate on this further here.

### 3.3 Other Diseases

In the central nervous system, DKK1 is associated with the pathophysiology of neuronal degeneration in Alzheimer’s disease (Caricasole et al., 2004; Vallée and Lecarpentier, 2016; Ren et al., 2019). DKK1 expression was found to be elevated in cortical neurons exposed to the β-amyloid peptide (β-AP), where its aberrant expression was responsible for hyperphosphorylation of tau in neurons challenged with β-AP. This was further validated by the observation of potentiated DKK1 expression in degenerating neurons in brain samples of Alzheimer’s disease patients (Caricasole et al., 2004). Consistent with these findings, an association between DKK1 and neuronal death in cellular and animal models of excitotoxic/ischemic neuronal death has been reported (Iozzi et al., 2012). DKK1 is also involved in placental lipid metabolism through the canonical Wnt pathway, with significantly increased β-catenin accumulation in obesity-prone placentas compared to obesity-resistant placentas (Strakovsky and Pan, 2012). Circulating DKK1 levels are high in type 2 diabetes mellitus, and a close relationship of DKK1 with cardiovascular disease has been revealed (Garcia-Martin et al., 2014; Zhang et al., 2015b). Indeed, serum DKK1 level has been shown to be negatively correlated with coronary atherosclerosis, which enables its use to predict coronary artery calcification. Nevertheless, the development of drugs targeting DKK1 for prevention of atherosclerosis-related diseases still requires more evidence and further exploration (Kim et al., 2011). Some available data also indicate that DKK1 is crucially involved in fibrosis, and its clear suppressive function makes it a feasible and attractive target in fibrotic diseases (Klavdianou et al., 2017).

## 4 DKK1 Inhibitors

### 4.1 Small Molecule Inhibitors

#### 4.1.1 NCI8642 (Gallocyanine)

**FIGURE 2:**
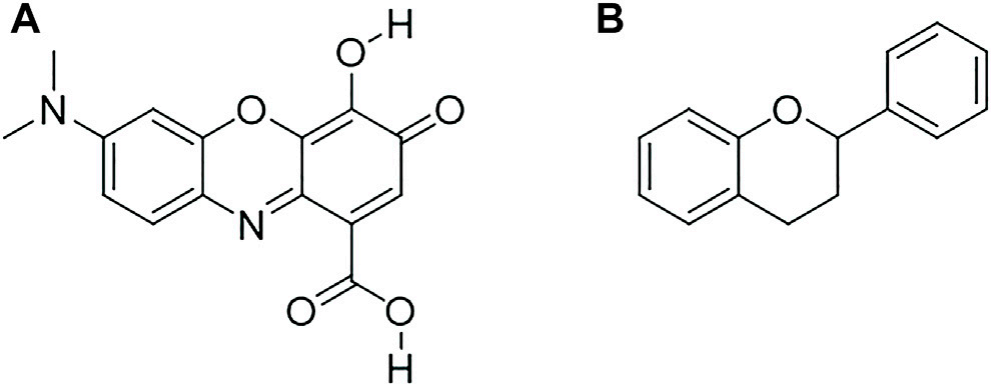
The 2D structure of small-molecule NCI8642 **(A)** and flavonoids **(B)**.

Wu et al. (2014a) virtually screened several compounds that could disrupt the crosstalk between LRP5 and DKK1 using the National Cancer Institute (NCI) database, including NCI106164, NCI39914, and NCI660224. This resource is freely searchable and contains more than 250,000 small chemical compounds. Owing to the common anthra-9, 10-quinone substructure found in NCI39914 and NCI660224, screening based on substructure helped to identify another two compounds, NCI366218 and NCI8642. Biological assays showed that NCI8642 was the best compound for disrupting the interplay of DKK1 with LRP5/6 and reversing the Wnt signaling mediated by DKK1 (Wu et al., 2014a; Klavdianou et al., 2017). NCI8642 could efficiently displace DKK1 from LRP5/6 and impede DKK1 inhibitory activity on canonical Wnt signaling, as demonstrated by binding and cellular assays, respectively. In the presence of exogenously added DKK1, NCI8642 could reverse DKK1-mediated inhibition; the effect was concentration-dependent and significantly distinct from that of dimethyl sulfoxide treatment at 25 and 50 μM. Although the micromolar potency of NCI8642 might be too low to enable its *in vivo* use, this compound could serve as a reference small molecule for further drug development aiming to inhibit therapeutically important protein-protein interactions (Iozzi et al., 2012). The above studies demonstrate that the DKK1-LRP5/6 interplay can be targeted by small molecules, which unlocks the possibility of new therapeutic tools for diseases associated with DKK1 dysregulation. However, the exact mechanisms by which these compounds restrain the function of DKK1 in the Wnt signaling pathway remain to be fully clarified (Glantschnig et al., 2010). It is found that epimedium-derived flavonoids with multiple benzene rings were also capable of downregulating DKK1 expression (Figure 2). This basic structure could thus have an important function in inhibiting DKK1 and is a potential candidate for accelerated virtual screening (Xu et al., 2011).

### 4.2 Biomacromolecule Inhibitors

#### 4.2.1 BHQ880

BHQ880 (Novartis, Cambridge, MA, United States) is a phage-derived DKK1 antibody that neutralizes both human DKK1 and murine DKK1 but does not show high affinity for DKK2 or DKK3. In the SCID-hu murine model, osteoblast number and function, serum human osteocalcin levels, and trabecular bone were significantly increased after administration of BHQ880, although there was no direct effect on MM cell growth. Nonetheless, BHQ880 inhibited the growth of MM cells in the presence of bone marrow stromal cells (BMSCs) *in vitro*, as demonstrated by significantly lower cell adhesion and IL-6 levels in the presence of BMSCs. In addition, β-catenin levels were upregulated under the regulation of BHQ880, with a simultaneous decrease in nuclear factor-κB activity in BMSCs (Fulciniti et al., 2009). Another *in vivo* study reported obvious anti-osteolytic effects of BHQ880 in the 5T2MM murine model of myeloma, with concurrent protection against 5T2MM-induced trabecular bone loss, but BHQ880 treatment to inhibit DKK1 did not reduce tumor burden (Heath et al., 2009). However, a study using a mouse model of subcutaneous osteosarcoma xenografts, aiming to determine the linkage between tumor growth velocity and DKK1 level, reported a statistically significant decrease in tumor growth velocity in mice treated with BHQ880 compared with control mice. In an orthotopic implantation/amputation model of osteosarcoma metastasis, BHQ880 was found to activate the Wnt pathway and attenuate metastasis, promoting differentiation and slowing tumor growth. Additionally, BHQ880 suppressed the growth and metastasis of orthotopically implanted patient-derived osteosarcoma xenografts, which was relevant to the increase of nuclear β-catenin staining and an elevated level of osteopontin (Goldstein et al., 2016). To evaluate the safety and efficacy of BHQ880, Padmanabhan et al. (2009). carried out a phase IB multicenter dose-determination study. The results showed no dose-limiting toxicity in the combination treatment with anti-myeloma therapy and zoledronic acid, but 96.4% of patients reported adverse events (AEs), although only one-third of these might have been related to BHQ880 or zoledronic acid (ZA). No grade 3–4 AEs or severe AEs related to the treatment were reported; the most common AEs were grade 1–2 treatment-emergent effects such as arthralgia, fatigue, and pyrexia (Munshi et al., 2012). Of three patients who received 24 cycles of treatment, one had stable disease and the other two had partial responses, and 75% of the patients had an active skeletal-related event at baseline. Overall, although studies in a larger patient population are required to confirm the efficacy of BHQ880 monotherapy, BHQ880 shows clinical potential for treating MM (Iyer et al., 2014). As well as its use in MM, BHQ880 was shown to inhibit bone metastases and tumor growth in prostate cancer, indicating that BHQ880 treatment could be an effective therapy for advanced prostate carcinoma (Sottnik et al., 2012).

#### 4.2.2 DKN-01

DKN-01 is a humanized IgG4 monoclonal antibody that neutralizes human and murine-derived DKK1 and specifically recognizes the C-domain of DKK1. A study of kinetic parameters showed that the *K*_D_ towards DKK1 of clinical DKN-01 was 28 pmol/L, similar to that of mouse-derived mDKN-01 (21 pmol/L) (Haas et al., 2021). Given the high expression and close involvement of DKK1 in MM, DKN-01 has been evaluated in patients with MM in preclinical studies and phase I testing. The data showed that DKN-01 possessed indirect anti-MM effects secondary to inhibition of osteoclastogenesis (Pozzi et al., 2013). In concordance with this, mDKN-01 exhibited no direct antiproliferative effect on B16F0 tumors, but a lack of DKK1 expression was detected after treatment (Haas et al., 2021). In an ovarian cancer mouse model, DKN-01 effectively modulated antitumor immunity, increasing natural killer (NK) cell infiltration (*p* = 0.045) and NK PD-L1 expression (*p* = 0.001), although there was no evident improvement in the survival outcomes (Betella et al., 2019). A mechanistic study demonstrated that DKN-01 required NK cells to exert its role in a syngeneic tumor model, which also illustrated the importance of NK cells in the efficacy of DKN-01 (Haas et al., 2019). In a phase I study of patients with advanced NSCLC, the median OS was 6.6 months after treatment with DKN-01, and the median progression-free survival was 2.2 months. No dose-limiting toxicities or serious AEs were found (Edenfield et al., 2014). An ongoing phase II basket study on patients with recurrent gynecologic cancer also confirmed that DKN-01 showed increased activity in combination therapy, but other correlative data in terms of safety and efficacy are still pending (Arend et al., 2019a).

#### 4.2.3 RH2-18

Novel fully human monoclonal antibodies including RH2-18, RH2-31, RH2-59, RH2-80, and RH2-18LC01 have been discovered through screening with a phage-displayed scFv library. The anti-DKK1 neutralizing antibody RH2-18 possesses excellent affinity for DKK1, with a mean *K*_D_ value reaching 249 pM. Epitope mapping experiments showed that these antibodies recognize conformational epitopes rather than specific DKK1 sequences. To be specific, they recognize only non-denatured native DKK1 (Glantschnig et al., 2010). To map the key DKK1 domains for functional interplay with these antibodies, deletion constructs and Ala substitution mutants were obtained. The results suggested that two continuous protected fragments, Val188-Lys217 and Cys239-Cys245 in the C-domain, were the interacting sites (Arend et al., 2019a). The function of RH2-18 in interfering with Wnt signaling by blocking DKK1 was validated in human embryonic kidney 293 cells and mouse pluripotent mesenchymal cells; RH2-18 could mediate the restoration of Wnt-induced activities (Glantschnig et al., 2011). Furthermore, although an obvious neutralizing effect appeared at an antibody concentration of 8 nM, the neutralization of the suppressive effect of recombinant human DKK1 on cell differentiation was dose-related. Generally, the half-life (t1/2) of RH2-18 was 12–19 days, and pharmacokinetic analyses in rhesus monkeys showed an extended half-life compared with that in lower species, demonstrating its applicability in non-human disease models. Modulation of DKK1 function through an experimental fully human anti-DKK1 antibody exhibited translational potential as a bone-anabolic agent for the treatment of human low bone mass disease, a characteristic of postmenopausal osteoporosis. When RH2-18 was injected subcutaneously into ovariectomized mice and rhesus macaques, the BMD was obviously increased, especially the trabecular bone BMD, and the bone microarchitecture in the mouse vertebra was improved. In addition to exerting function on the axial skeleton, integral volumetric BMD, along with trabecular thickness at the distal radius and tibia, was significantly ameliorated as well after the administration of RH2-18 (Glantschnig et al., 2010; Glantschnig et al., 2011).

#### 4.2.4 Hetero-DS (Bispecific Antibody)

Owing to the compensatory effects of DKK1 levels and sclerostin expression, and the therapeutic potential of antibodies that target sclerostin (Scl-Ab) and DKK1 (DKK1-Ab) in treating bone disorders, including for improving fracture healing and increasing bone formation, Florio et al. (2016) proposed that a synergistic effect was triggered by the simultaneous blockage of DKK1 and sclerostin. Through hybridizing heavy chains and light chains of human Scl-Abs and human DKK1-Abs, a human sclerostin and DKK1 bispecific antibody (hetero-DS) was created. It had a K_D_ of 42 and 702 pM towards sclerostin and DKK1, respectively, and neutralized DKK1 and sclerostin with a similar potency to parental antibodies. Both fracture repairing experiments in rats and bone-forming effects experiments in primates showed that hetero-DS possessed more robust therapeutic efficacy than the parental antibodies (Florio et al., 2016). Furthermore, blocking DKK1 not only rescued the deterioration in osteoblastogenesis but neutralized TNF-mediated sclerostin expression in entirely differentiated osteoblasts *in vitro* and *in vivo*, resulting in stronger activation of the Wnt pathway. This makes concomitant targeting DKK1 and sclerostin a more promising approach to treating bone diseases relative to either monotherapy (Heiland et al., 2010). To date, no other study has reported the development of a bispecific antibody binding DKK1 (Table 1). However, the method for constructing bispecific antibodies will aid the future study of antibodies. For example, the development of multispecific antibodies combining the advantages of several antibodies is a faster route to overcome their disadvantages, especially when the defective monospecific antibodies target the same proteins.

**TABLE 1:** Development of antibodies against DKK1.

Antibodies	Model(s)	Disease(s)	References
BHQ880	5T2MM murine model of myeloma	MM	Heath et al. (2009)
	SCID-hu mouse model of human myeloma	MM	Fulciniti et al. (2009)
	Orthotopic patient-derived xenograft model of osteosarcoma	Osteosarcoma	Goldstein et al. (2016)
DKN-01	Patients with relapsed/refractory esophagogastric cancer	Esophagogastric cancer	Wall et al. (2020)
	Patients with biliary tract cancer	Biliary tract cancer	Goyal et al. (2020)
	C57BL/6 mice	Ovarian cancer	Betella et al. (2019)
	ID8 syngeneic mice	Epithelial ovarian cancer	Betella et al. (2020)
	Patients with MM or advanced solid tumors	MM or advanced solid tumors	Ryan et al. (2016)
	Gynecologic malignancy patients	Gynecologic malignancies	Arend et al. (2019a)
	NSCLC patients	NSCLC	Edenfield et al. (2014)
RH2-18	Ovariectomized C57BL/6NTac mice and rhesus macaques	Postmenopausal osteoporosis	Glantschnig et al. (2011)
Hetero-DS	Femur fracture model in rats	Fracture	Florio et al. (2016)
	Cynomolgus monkey	Postmenopausal osteoporosis	Florio et al. (2016)

#### 4.2.5 Cyclized Oligopeptide

Park et al. (2017) synthesized a cyclized oligopeptide consisting of 10 amino acids, with the amino acid composition Cys-Asn-Ser-Asn-Ser-Ile-Arg-Phe-Cys-Gly. This oligopeptide contained the Asn-X-Ile (NXI) motif that appears to be conserved in DKK1 (Park et al., 2017). As reported previously, the NXI motif closely matches the E1 domain of LRP5/6, which is critical in the binding between DKK1 and LRP5/6 (Bourhis et al., 2011). The cyclized oligopeptide specifically inhibited the DKK1-LRP5/6 interaction, but it did not abrogate the inhibitory effect of sclerostin on Wnt signaling, irrespective of the existence of a similar NXI motif in sclerostin. In an *in vitro* experiment, this oligopeptide presented bone-anabolic effects. To evaluate its effects on cancer cells *in vivo*, the MOPC315. BM.Luc MM mouse model was adopted. Treatment with this oligopeptide significantly reduced the tumor burden, whereas MM cell growth was not influenced directly. Although its mechanism of action on osteoblasts and MM cells remains uncertain, this oligopeptide sequesters DKK1 from LRP5/6 and reduces tumor cell growth, providing an option for managing tumor burden in MM (Park et al., 2017).

#### 4.2.6 Other Biomacromolecules

In addition to anti-DKK1 antibodies, endothelin-1 (ET-1), an endothelium-derived vasoconstrictor that mediates osteoblastic bone metastases, was found to target DKK1 and elicit responses by osteoblasts (Vaiou et al., 2016). When mouse neonatal calvariae were treated with ET-1, DKK1 mRNA levels and protein secretion were significantly decreased, and a robust increase in new bone formation was observed (Clines et al., 2007). Nonetheless, ET-1 can trigger resistance to bortezomib in human MM cells, and it has been suggested that ET-1 might be a key factor involved in the escape mechanism in MM patients treated with bortezomib (Vaiou et al., 2016). Another type of inhibitor suppresses DKK1 by inhibiting DKK1 mRNA levels, *via* a mechanism similar to that of microRNAs (miRNAs). For instance, protein arginine methyltransferase 5 binds to the promoter region of DKK1 and DKK3, and symmetrically methylates the H3R8 and H4R3 histones, achieving epigenetic silencing of DKK1 (Shailesh et al., 2021).

### 4.3 Nucleic Acid Inhibitors

#### 4.3.1 Anti-Sense Oligonucleotides

Anti-sense oligonucleotides are small pieces of DNA or RNA that hybridize to target molecules of RNA, blocking the transcription of RNA into protein by preventing ribosome recruitment and thereby modulating specific protein expression (Muntoni and Wood, 2011; Rinaldi and Wood, 2018). As a novel therapeutic intervention, antisense oligonucleotides have actually been widely utilized to inhibit various disease-related gene expressions. They are easy to make or modify and have shown higher success rates in targeting the source of the pathogenesis than drugs targeting downstream pathways (Baker et al., 1997; Leech et al., 2000). End-capped phosphorothioate DKK1 antisense oligonucleotides (DKK1-AS) include DKK1-AS1 and DKK1-AS2, which are complementary to nucleotides 4–21 and 243–260 of DKK1 mRNA respectively. They were utilized to alleviate DKK1 expression in osteoarthritic knees. A resultant significant decrease in DKK1 expression was observed, and the decline in BMD was considerably reversed (Weng et al., 2010; Weng et al., 2012; Wijesinghe et al., 2021). Before this, DKK1-AS had been found to abrogate loss of bone mass and biomechanical properties in ovariectomized rats; the regulatory mechanism seems to be the suppression of the ovariectomy-promoted osteoclastogenesis-regulatory activities of bone cells (Wang et al., 2007). This suggests that controlling DKK1 signaling using antisense oligonucleotides might be an alternative strategy in treating postmenopausal osteoporosis. To exclude the implication of DKK1 in kainate-induced seizures, Busceti et al. (2007) injected DKK1-AS into adult male Sprague-Dawley rats to achieve the knockdown of DKK1. The antisense treatment not only prevented DKK1 expression but also neuronal death in the olfactory cortex and hippocampus at 3 days (Busceti et al., 2007). Consistently, treatment with DKK1-AS prevented ischemic injury to hippocampal neurons and N-methyl-d-aspartate toxicity from cultured cortical neurons, thus demonstrating the great importance of DKK1 in the development of ischemic and excitotoxic neuronal death (Cappuccio et al., 2005). Taken collectively, these data demonstrate that rescuing Wnt signaling by DKK1 anti-sense oligonucleotides has therapeutic potential at least for treating bone diseases.

#### 4.3.2 Small Interfering RNAs (siRNAs)

RNA interference (RNAi) is a prominent biological process of post-transcriptional gene silencing mediated by specific double-stranded RNA sequences, which turns off the expression of related genes (Tijsterman and Plasterk, 2004). After the enzymatic digestion of the input double-stranded RNA by DICER, the resulting siRNAs destruct the expression of homologous mRNA and achieve the effect of RNAi (Xia et al., 2002). Feng et al. (2019). reported that the transfection of DKK1-siRNA into U266 cells significantly decreased DKK1 expression relative to that in the control group, simultaneously reducing the activity of U266 cells and IL-6 expression. More significantly, transfecting MDA-MB-231 cells with DKK1 siRNA completely reversed the NBAT1-induced upregulation of DKK1 (Zhang et al., 2019). In an *in vivo* mouse model experiment, treatment with siRNA was capable of increasing osteocalcin content and stimulating osteoblast differentiation (Feng et al., 2019). Silencing DKK1 expression by transfecting a siRNA targeting human DKK1 (Hs_DKK1_1) rescued the dexamethasone-induced inhibition of osteoblast differentiation in primary human osteoblasts (Butler et al., 2010). Some pieces of evidence showed that when positive regulatory domain I (PRDM1) was overexpressed, DKK1 expression was unexpectedly enhanced. To determine that PRDM1 is independent of DKK1 regulation in glioma cells, siRNA was introduced for the purpose of silencing DKK1 expression. There were no side-effects resulting from treatment, which highlighted the biosafety of siRNA (Wang et al., 2013). In addition, siRNA was found to substantially downregulate DKK1 expression in esophageal cancer cells, which contributed to a decrease in abnormal cell proliferation (Yi et al., 2013). Therefore, siRNA-mediated RNAi targeting DKK1 might be a promising therapy for treating esophageal carcinoma (Lu et al., 2017b). Indeed, studies so far have merely used DKK1 siRNA as a tool for gene silencing and applied it to diverse disease models; thus, the corresponding drug development has a long way to go.

#### 4.3.3 MiRNAs

As highly conserved non-coding RNAs of approximately 17–25 nucleotides, miRNAs regulate the expression of target genes by binding to the 3′-UTR (untranslated region) (Inui et al., 2010). MiRNAs have been extensively studied in the past few years, and DKK1 expression has been found to be regulated by a variety of miRNAs (Supplementary Table S1). In oral squamous cell carcinoma (OSCC) cells, miR-1-3p suppressed the progression of OSCC by attenuating DKK1 expression and thereby triggering apoptosis, which implied its potential for cancer treatment (Wang et al., 2018c). MiR-29a and miR-335-5b are also effective regulators of DKK1 in osteoblasts, and there might be some complementarities between them and DKK1 mRNA (Kapinas et al., 2010; Zhang et al., 2011). Similarly, miR-203 showed regulatory effects on osteoblasts, but it could also further reverse the osteogenic differentiation in mesenchymal stem cells and participate in the pathogenesis of osteoporosis by binding to DKK1 (Xia et al., 2018). In osteosarcoma and adjacent tissues, DKK1 expression was negatively correlated with miR-107 (*p* = 0.01). By introducing mutations at predicted miR-107-binding sites, it was proved that the transcription of DKK1 could be hindered by binding of miR-107 to the 3′-UTR of DKK1 (Zhang et al., 2017). In colon cancer cells and HCC tissues, miR-217 directly interacted with the 3′-UTR of DKK1 and diminished DKK1 expression, constitutively activating Wnt signaling and promoting the tumorigenesis and progression of HCC. In turn, DKK1 overexpression antagonized the stimulatory role of miR-217 in Wnt signaling, further indicating the importance of miR-217 in HCC cells (Jiang et al., 2017; Jia et al., 2019). In mouse embryonic stem cells, overexpression of miR-290 resulted in lower expression of DKK1 and promoted cluster pluripotency against differentiation (Zovoilis et al., 2009). Likewise, DKK1 was identified as a direct target of miR-372 and miR-373 in breast cancer and CRC cells, and knockdown of DKK1 promoted the growth and invasive activity of cancer cells (Zhou et al., 2012). Many other miRNAs, in addition to the ones mentioned above, have been identified as related to DKK1, including miR-9, miR-31, miR-33a-5p, miR-34a, miR-92b, miR-101, miR-103a-3p, miR-130b-3p, miR-152, miR-291a-3p, miR-302b, miR-373-3p, miR-410, miR-433-3p, miR-488, miR-493, miR-501-5p, miR-522, miR-523-3p, miR-543, miR-590-3p, miR-613, miR-3064-3p, and miR-BART10-3p (Liu et al., 2016b; Fan et al., 2016; Jia et al., 2016; Zhang et al., 2016; Feng et al., 2017; Lv et al., 2017; Tang et al., 2017; Weng et al., 2017; Wang et al., 2018a; Wang et al., 2018b; Chen et al., 2018; Zhao et al., 2018; Liu et al., 2019; Min and Lee, 2019; Wang et al., 2019; Li et al., 2020a; Li et al., 2020b; Liu et al., 2020; Xiang et al., 2020; Zeng et al., 2020; Zhou et al., 2020; Liao et al., 2021; Song et al., 2021; Wu et al., 2021).

#### 4.3.4 Dickkopf-1 Vaccines

DNA vaccination is a new approach to directly induce humoral and/or cellular immune responses without live vectors. For the generation of DNA vaccines, the antigen-encoding gene is inserted into a plasmid with an appropriate eukaryotic promoter, and the obtained recombinant plasmids are then administrated to animals for the production of antigens, which will elicit immune responses (Lu et al., 2008; Hasson et al., 2015). To determine whether the immunity elicited by a DKK1 vaccine could alleviate myeloma, a DNA (murine DKK1/defensin-2 fusion) vaccine was employed in the murine MOPC-21 myeloma model. Although only three of ten mice injected with the DNA vaccine survived in prophylactic studies, the percentage of mice with no tumor burden increased to 60% after treatment with the DNA vaccine plus adjuvant CpG. In the tumor inhibition experiment, the therapeutic efficacy was remarkable (Table 2). Concurrently, CD^4+^ and CD^8+^ T cells in vaccinated mice were activated, resulting in stronger tumor-specific responses compared with those of the mice without vaccination (Qian and Yi, 2012; Liu et al., 2018). Zhang et al. (2015a) constructed a multiepitope DNA vaccine against DKK1 by screening and optimizing four B cell epitopes; the results implied that vaccination could attenuate bone erosion relevant to DKK1 in collagen-induced arthritis (CIA) mice and lower the disease incidence of arthritis in CIA-susceptible mice. To further facilitate the efficiency of the DNA vaccine and circumvent the limitations of the conventional DNA-immunization approach, a novel hybrid phage particle was used to carry the DKK1 gene (Khalili et al., 2018). The phage-immunization results showed higher efficiency of the immunization vehicle and stronger humoral immune responses in contrast to classical DNA-immunization vectors, which meant that larger amounts of antibodies against DKK1 were produced. By means of combining epitopes originating from MM special antigen-1 (MMSA-1) and DKK1, Lu et al. (2017a) constructed a multi-epitope peptide vaccine that improved the immune response and attenuated myeloma growth. Indeed, peptide vaccines have presented impressive potential in provoking immune reactions and eliminating cancer cells (Perez et al., 2010; Walz et al., 2015). Wu et al. (2014b). explored the inhibitory effects of DKK1-derived peptides on cell differentiation and bone metabolism in MC3T3-E1 pre-osteoblastic cells and found increased bone mass, improved bone microstructure, reduced bone loss, and decreased mineralization in mice vaccinated with DDPs. Remarkably, the serum anti-DKK1 antibody titers were distinctly increased, with augmentation of more than 100,000 times compared with average titers 6 weeks after the first injection. Therefore, whether it is better to develop DNA vaccines or other traditional vaccines depends on the final clinical effects (Wu et al., 2014b).

**TABLE 2:** The effects of different DKK1 inhibitors in multiple models.

DKK1 inhibitors		Regulatory activities	References
Small molecules	NCI8642	NCI8642 reversed the DKK1-mediated inhibition of Wnt signaling, but its effect was concentration-dependent	Iozzi et al. (2012)
Biomacromolecules	BHQ880	In the SCID-hu murine model, BHQ880 inhibited the growth of MM cells in the presence of BMSCs *in vitro*, and it also showed anti-osteolytic effects in the 5T2MM murine model of myeloma. In addition, BHQ880 could activate the Wnt pathway, attenuate metastasis, promote differentiation, and slow tumor growth in an orthotopic implantation/amputation model of osteosarcoma metastasis	Fulciniti et al. (2009), Heath et al. (2009), Goldstein et al. (2016)
	DKN-01	DKN-01 showed indirect anti-MM effects in preclinical studies and phase I testing, and DKN-01 treatment prolonged the median OS in patients with advanced NSCLC	Pozzi et al. (2013), Edenfield et al. (2014)
	RH2-18	RH2-18 could restore Wnt-induced activities, and BMD was increased in ovariectomized mice and rhesus macaques	Glantschnig et al. (2010); Glantschnig et al. (2011)
	Hetero-DS	Hetero-DS promoted bone mass accrual and fracture repair, and it showed more robust therapeutic efficacy than parental antibodies (Scl-Ab and DKK1-Ab)	Heiland et al. (2010), Florio et al. (2016)
	Cyclized Oligopeptide	An oligopeptide showed bone-anabolic effects *in vitro* and significantly reduced the tumor burden in the MOPC315.BM.Luc MM mouse model	Park et al. (2017)
Nucleic acids	Anti-sense oligonucleotides	Several anti-sense oligonucleotides have been used to inhibit DKK1 expression *in vivo*, and loss of bone mass was abrogated in ovariectomized rats after treatment	Wang et al. (2007), Weng et al. (2010), Weng et al. (2012)
	SiRNAs	Transfection of DKK1-siRNA into cells and mice could attenuate DKK1 expression, thereby stimulating osteoblast differentiation or decreasing abnormal cell proliferation in different models	Butler et al. (2010); Feng et al. (2019)
	MiRNAs	MiRNAs inhibit DKK1 expression by directly binding to the 3′-UTR of DKK1 mRNA and have been applied in various cancer models	Zovoilis et al. (2009), Jiang et al. (2017), Wang et al. (2018c)
	DKK1 vaccines	DKK1 vaccines helped to elicit immune responses to alleviate myeloma in the murine MOPC-21 myeloma model, with remarkable therapeutic efficacy in tumor inhibition experiments. Furthermore, a multiepitope DKK1 vaccine could attenuate bone erosion and lower the disease incidence of arthritis in CIA mice	Qian and Yi (2012); Zhang et al. (2015a)

#### 4.3.5 Other Nucleic Acids

Long noncoding RNA (lncRNA) LINC01133 was shown to methylate the DKK1 promoter, and its upregulation was associated with increased DKK1 methylation and decreased DKK1 expression in pancreatic cancer (Weng et al., 2019). Another lncRNA, HOTTIP, also mediated DKK1 downregulation in CRC cells (Rui et al., 2018). Two genes with longer sequences, muscle segment homeobox 1 (MSX1) and GATA6, suppressed DKK1 transcription by directly binding to the DKK1 promoter (Zhong et al., 2011; Menezes et al., 2012). Likewise, c-Myc, which is a frequently amplified gene in breast cancers, suppressed DKK1 and transformed human mammary epithelial cells (Cowling et al., 2007).

## Conclusion and Perspectives

**FIGURE 3:**
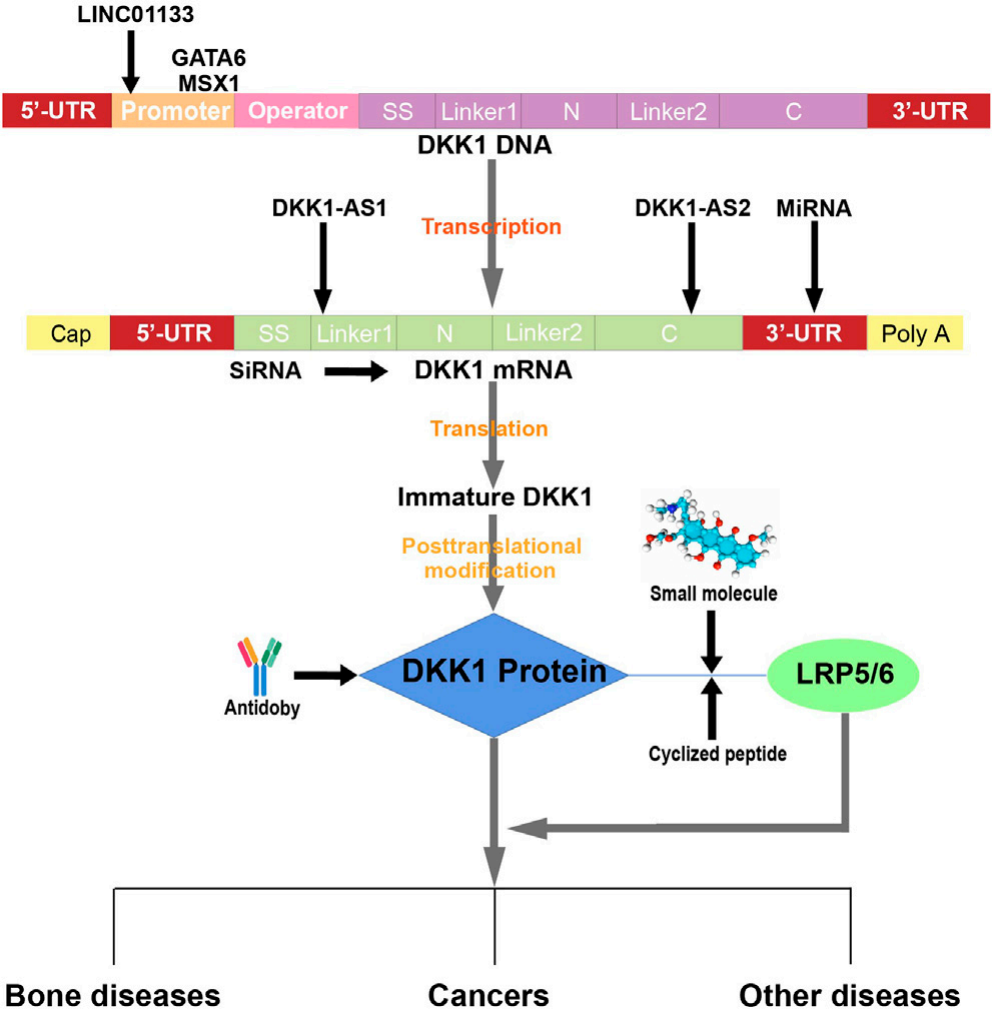
Diagram of inhibition by DKK1 inhibitors. LINC01133 methylates the DKK1 promoter, but GATA6 and MSX1 directly bind to the DKK1 promoter. After the transcription of DKK1 DNA, DKK1-AS1 and DKK1-AS2 bind to nucleotides 4–21 and 243–260 of DKK1 mRNA, respectively. MiRNAs bind to the 3′-UTR of DKK1 mRNA, but siRNAs bind to the translational region. DKK1 antibodies specifically interact with DKK1 protein, but different antibodies bind to distinct domains of DKK1, and a cyclized oligopeptide competitively binds with LRP5/6 and therefore blocks the DKK1-LRP5/6 interaction because it has the same NXI motif as the DKK1 protein. NCI8642 is a small molecule that can function as a DKK1 inhibitor by suppressing the DKK1-LRP5/6 interaction.

Anti-DKK1 antibodies are the most intensively studied of the current DKK1 inhibitors. Although many studies have demonstrated the potent effectiveness of several antibodies against DKK1, some limitations have emerged in preclinical and clinical trials. For instance, BHQ880 treatment significantly decreased osteosarcoma growth velocity in mice at the early phase; however, this effect vanished when tumors were in the advanced stage or serum DKK1 could not be detected (Goldstein et al., 2016). Furthermore, BHQ880 was primarily characterized and applied in MM models, with only a few publications reporting its function in other diseases. Unlike BHQ880, DKN-01 has been reported to have effects in various cancers, including NSCLC, biliary cancer, esophageal cancer, and gastroesophageal junction tumor, although the precise performance and related mechanism are still being investigated (Ryan et al., 2016; Arend et al., 2019a; Arend et al., 2019b). Nevertheless, mDKN-01 alone was unable to alter survival outcome, omentum tumor weight, or tumor burden in ovarian cancer (Betella et al., 2020). Moreover, the efficacy of mDKN-01 in a syngeneic tumor model was revealed to require NK cells, which means that the immunotherapeutic effects of mDKN-01 could be suppressed if NK cells were damaged or killed in certain tumors (Haas et al., 2019). Research on RH2-18 and hetero-DS has so far only investigated their effects on bone disorders; no other successful attempt has been published. Small molecules and nucleic acid inhibitors played a minor part in the present review, as almost all ongoing clinical trials of DKK1 inhibitors involve monoclonal antibodies. Although the development of antibodies is pivotal to both human health and commercial applications, different types of drugs should be considered that can compensate for each other to provide patients with the best treatment effects (Figure 3).

Combination therapy is a well-known and effective way to overcome the drawbacks of monotherapy and strengthen therapeutic effects. To date, preclinical or clinical tests have shown that several DKK1 inhibitors can cooperate with natural products or antibodies to achieve better efficacy. The therapeutic antibody BHQ880 was well tolerated in combination with an anti-MM therapy and ZA, and BMD increased over time, although the contribution of BHQ880 was not established (Iyer et al., 2014). DKN-01 in combination with paclitaxel in patients with advanced esophageal cancer or gastroesophageal junction tumors enhanced activity relative to paclitaxel monotherapy, with no alteration in safety profile (Ryan et al., 2016). Similarly, an mDKN-01/anti-PD-1 combination showed better efficacy in inhibiting melanoma growth than mDKN-01 alone, and DKN-01 + pembrolizumab also optimized safety and clinical activity in advanced gastroesophageal adenocarcinoma (Haas et al., 2021). A phase I study on combination therapy with gemcitabine and cisplatin in patients with advanced biliary cancer showed that DKN-01 was tolerated well and stable disease could be possibly prolonged; potential antiangiogenic and immunomodulatory activity could be introduced by DKN-01 (Eads et al., 2016; Goyal et al., 2020). In preclinical ovarian cancer models, DKN-01 synergistically coordinated anti-tumor immunity with other drugs and increased the sensitivity of ovarian cancer cells to immune-checkpoint drugs, thereby laying the foundation of a promising combinatorial therapy for ovarian carcinoma (Betella et al., 2020). Other small molecules such as bortezomib combined with dexamethasone facilitated the response rates of relapsed or refractory MM patients and were well tolerated in clinical trials, although DKK1 was not their target (Jagannath et al., 2006; Ozaki et al., 2007). Collectively, all of these results highlight the great prospects and potential of combination therapy in clinical applications. This topic is expected to receive considerable attention in the future.

Therefore, on the basis of the known structural information about DKK1 and the combination of bioinformatics tools available, screening specific small molecules from massive databases is a viable and effective strategy. At present, the discovery of DKK1 inhibitors is still in its infancy, particularly in the case of small-molecule inhibitors. Following the finding that NCI8642 can block DKK1-LRP6 interaction, other chemicals exclusively binding to a specific domain of DKK1 should be the focus of further research. For instance, the C-domain of DKK1 is necessary for the interplay between DKK1 and LRP6, and some key sites have been identified. Through virtually screening compounds interacting with these residues and subsequent experimental verification, effective candidates might be obtained after screening a library of compounds. Accordingly, with the assistance of computational tools, the drug discovery of next-generation DKK1 inhibitors could be accelerated. Regarding other nucleic acid inhibitors, despite many reports of their inhibitory effects towards DKK1 expression in a variety of common cancer cells, no nucleic acid drug targeting DKK1 has yet been marketed to treat DKK1-associated diseases. With the gradual elucidation of nucleic-acid-related disease mechanisms and the advancement of drug delivery systems, significant achievements in clinical applications are expected. Aptamers, which are critical components of nucleic acid drugs, can be regarded as “chemical antibodies” owing to their high selectivity and specificity towards their targets, and their ease of access and more flexible recognition sites could further enable them to compete with antibodies (He et al., 2019; Chen et al., 2021). To date, only two DKK1 aptamers, obtained by Zhou et al., have been reported for use in early diagnosis of HCC; therapeutic aptamers are still lacking (Zhou et al., 2019). Therefore, there is an urgent need for the development of DKK1 aptamers, although DKK1 antibodies are currently preferentially evaluated and used clinically.
