# Supplementary material for: Drug Discovery of DKK1 Inhibitors
Source: Front Pharmacol. 2022 Mar 9;13:847387. doi: 10.3389/fphar.2022.847387 (PMC8959454; doi:10.3389/fphar.2022.847387)
Supplement: Supplementary file 1 [file Table1.DOCX]

Supplementary Material

**Table S1 The effects of miRNAs targeting DKK1 in various models.**

| **MiRNA** | **Model** | **Activities** | **Reference** |
| --- | --- | --- | --- |
| **MiR-1-3p** | Oral squamous cell carcinoma | MiR-1-3p suppresses the progression of OSCC by inhibiting DKK1 expression. | (Wang et al., 2018c) |
| **MiR-9** | Mesenchymal stem cell line C2C12 | MiR-9 promotes osteoblast differentiation of mesenchymal cell C2C12 by suppressing DKK1 expression. | (Liu et al., 2016) |
| **MiR-29a** | Human fetal osteoblastic 1.19 cell line | MiR-29a binds directly to the 3’-UTRs of DKK1, Kremen2, and sFRP2 and negatively regulates their expression. | (Kapinas et al., 2010) |
| **MiR-31** | HC11 mouse mammary epithelial cells  NOD-SCID mice with mammary glands and PyVT tumors | MiR-31 promotes mammary stem cell expansion and breast tumorigenesis by directly targeting Dkk1. | (Lv et al., 2017) |
| **MiR-33a-5p** | Esophageal cancer cell lines | MiR-33a-5p suppresses DKK1 expression to affect the biological behavior of esophageal cancer cells, such as cell proliferation, migration, invasion, and apoptosis. | (Song et al., 2021) |
| **MiR-34a** | Human bone marrow mesenchymal stem cells | MiR-34a specifically binds with DKK1, and ART could promote osteoblast differentiation through miR-34a/DKK1/Wnt pathway. | (Zeng et al., 2020) |
| **MiR-92b** | The trophoblast cell line | MiR-92b negatively regulates DKK1 expression and inhibits the migration and invasion of trophoblasts. | (Li et al., 2020a) |
| **MiR-101** | Human bone mesenchymal stem cell | DKK1 directly interacts with mir-101 and regulates osteogenic differentiation. | (Xiang et al., 2020) |
| **MiR-103a-3p** | Mesenchymal stem cells | MiR-103a-3p increases the expression levels of osteogenesis‑related proteins by targeting DKK1 expression. | (Liu et al., 2020) |
| **MiR-107** | Human osteosarcoma cells | MiR-107 promotes cancer cell apoptosis by inhibiting the protein expression of DKK1 and down-regulating the protein expression of Bcl-2. | (Zhang et al., 2017) |
| **MiR‑130b‑3p** | Melanoma cell lines | MiR-130b-3p inhibits DKK1 expression and represses ferroptosis in melanoma by directly binding to the DKK1 mRNA. | (Liao et al., 2021) |
| **MiR-152** | Human Osteosarcoma F5M2 cell line | MiR-152 targeting DKK1 can inhibit cell proliferation, induce apoptosis, and promote LDH activity, caspase-3/9 activity, Bax/Bcl-2, and p53 protein expression levels in osteosarcoma. | (Zhao et al., 2018) |
| **MiR-203** | Mesenchymal stem cells | MiR-203 could bind to DKK1 and promote the differentiation of rat MSCs into osteoblast-like cells. | (Xia et al., 2018) |
| **MiR-217** | Mepatocellular carcinoma cell lines  Colon cancer cell lines | MiR-217 promotes the CSC-like phenotype and regulates Wnt signaling via targeting DKK1. | (Jiang et al., 2017) |
| **MiR-290** | Mouse embryonic stem cells | MiR-290 cluster targets the DKK1 gene and favors pluripotency against differentiation. | (Zovoilis et al., 2009) |
| **MiR-291a-3p** | Bone marrow-derived mesenchymal stem cells | MiR-291a-3p promotes BMSCs' osteogenic differentiation by directly suppressing DKK1 mRNA and protein expression. | (Li et al., 2020b) |
| **MiR-302b** | Multiple myeloma cell lines | MiR-302b could target DKK1 and suppress cell proliferation and induce cell apoptosis. | (Wu et al., 2021) |
| **MiR-335-5p** | HEK 293 cells, MLO-A5 murine preosteocyte-like cells | MiR-335-5p activates Wnt signaling and promotes osteogenic differentiation by specifically targeting DKK1 3’-UTR. | (Zhang et al., 2011) |
| **MiR-372 / miR-373 / miR-373-3p** | Colorectal cancer cell lines  Tongue Squamous Cell Carcinoma | DKK1 is a direct target of miR-372 and miR-373, and the overexpression of miR-372 or miR-373 promotes cell growth. Moreover, miR-373-3p promotes the EMT-induced metastasis of tongue squamous cell carcinoma. | (Zhou et al., 2012)  (Weng et al., 2017) |
| **MiR-410** | Colorectal cancer | MiR-410 negatively regulates the expression of DKK1 and promotes malignancy phenotypes in CRC cell lines. | (Wang et al., 2019) |
| **MiR-433-3p** | Human osteoblast cells | MiR-433-3p promotes osteoblast differentiation through targeting DKK1 expression, and it can inhibit over 90% of DKK1 protein expression. | (Tang et al., 2017) |
| **MiR-488** | Mouse osteoblastic cell line | DKK1 is a direct target of miR-488, and the decreased expression of DKK1 improves cell viability and suppresses cell apoptosis. | (Wang et al., 2018b) |
| **MiR-493** | Gastric cancer cell lines | DKK1 is a novel direct target of miR-493 in GC cells, and miR-493 overexpression promotes the cell proliferation, invasion and chemo-resistance of GC cells. | (Jia et al., 2016) |
| **MiR-501-5p** | Gastric cancer cell lines | MiR-501-5p activates the Wnt signaling by directly targeting DKK1, NKD1 and GSK3β, thereby promoting the gastric cancer stem cell like phenotype. | (Fan et al., 2016) |
| **MiR-522** | Hepatocellular carcinoma | DKK1 is the target of miR-522, and miR-522 overexpression promotes cell proliferation, colony formation, and cell cycle progression. | (Zhang et al., 2016) |
| **MiR-523-3p** | Retinoblastoma cell lines | MiR-523-3p negatively regulates DKK1, and miR-523-3p downregulation can slow down cell growth, invasion, and migration and promote cell apoptosis in retinoblastoma cells. | (Zhou et al., 2020) |
| **MiR-543** | Renal cell carcinoma cell lines | DKK1 is a direct downstream target of miR-543, and miR-543 promotes cell proliferation and metastasis of renal cell carcinoma by targeting DKK1. | (Chen et al., 2018) |
| **MiR-590-3p** | Colon cancer cell lines | MiR-590-3p promotes colon cancer cell proliferation via the canonical Wnt signaling pathway by inhibiting WIF1 and DKK1. | (Feng et al., 2017) |
| **MiR-613** | Rheumatoid arthritis synovial fibroblasts | MiR-613 can inhibit proliferation and invasion and induce apoptosis of rheumatoid arthritis synovial fibroblasts by directly targeting DKK1 3’-UTR. | (Liu et al., 2019) |
| **MiR-BART10-3p** | Gastric carcinoma cells | MiR-BART10-3p regulates DKK1 and promotes cell proliferation and migration by directly targeting the 3’-UTR of DKK1 mRNA. | (Min and Lee, 2019) |
| **MiR-3064-3p** | Cementoblast cell lines | MiR-3064-3p suppresses the differentiation of cementoblasts by targeting DKK1. | (Wang et al., 2018a) |

**Reference**

Chen, Z.-y., Du, Y., Wang, L., Liu, X.-h., Guo, J., and Weng, X.-d. (2018). MiR-543 promotes cell proliferation and metastasis of renal cell carcinoma by targeting Dickkopf 1 through the Wnt/β-catenin signaling pathway. *J. Cancer* 9(20)**,** 3660. doi: 10.7150/jca.27124.

Fan, D., Ren, B., Yang, X., Liu, J., and Zhang, Z. (2016). Upregulation of miR-501-5p activates the wnt/beta-catenin signaling pathway and enhances stem cell-like phenotype in gastric cancer. *J. Exp. Clin. Cancer Res.* 35(1)**,** 177. doi: 10.1186/s13046-016-0432-x.

Feng, Z., Xu, X., Cen, D., Luo, C., and Wu, S. (2017). miR-590-3p promotes colon cancer cell proliferation via Wnt/beta-catenin signaling pathway by inhibiting WIF1 and DKK1. *Eur. Rev. Med. Pharmacol. Sci.* 21(21)**,** 4844-4852.

Jia, X., Li, N., Peng, C., Deng, Y., Wang, J., Deng, M., et al. (2016). miR-493 mediated DKK1 down-regulation confers proliferation, invasion and chemo-resistance in gastric cancer cells. *Oncotarget* 7(6)**,** 7044. doi: 10.18632/oncotarget.6951.

Jiang, C., Yu, M., Xie, X., Huang, G., Peng, Y., Ren, D., et al. (2017). miR-217 targeting DKK1 promotes cancer stem cell properties via activation of the Wnt signaling pathway in hepatocellular carcinoma. *Oncol. Rep.* 38(4)**,** 2351-2359. doi: 10.3892/or.2017.5924.

Kapinas, K., Kessler, C., Ricks, T., Gronowicz, G., and Delany, A. M. (2010). miR-29 modulates Wnt signaling in human osteoblasts through a positive feedback loop. *J. Biol. Chem.* 285(33)**,** 25221-25231. doi: 10.1074/jbc.M110.116137.

Li, N., Huang, L., Li, Y., Chen, X., Yang, Y., Hou, Y., et al. (2020a). Lin28B/miR-92b Promote the Proliferation, Migration, and Invasion in the Pathogenesis of Preeclampsia via the DKK1/Wnt/β-Catenin Pathway. *Reprod. Sci.* 27(3)**,** 815-822. doi: 10.1007/s43032-019-00083-8.

Li, Z. H., Hu, H., Zhang, X. Y., Liu, G. D., Ran, B., Zhang, P. G., et al. (2020b). MiR-291a-3p regulates the BMSCs differentiation via targeting DKK1 in dexamethasone-induced osteoporosis. *Kaohsiung J. Med. Sci.* 36(1)**,** 35-42. doi: 10.1002/kjm2.12134.

Liao, Y., Jia, X., Ren, Y., Deji, Z., Gesang, Y., Ning, N., et al. (2021). Suppressive role of microRNA-130b-3p in ferroptosis in melanoma cells correlates with DKK1 inhibition and Nrf2-HO-1 pathway activation. *Hum. Cell* 34(5)**,** 1532-1544. doi: 10.1007/s13577-021-00557-5.

Liu, J., Wu, M., Feng, G., Li, R., Wang, Y., and Jiao, J. (2020). Downregulation of LINC00707 promotes osteogenic differentiation of human bone marrow‑derived mesenchymal stem cells by regulating DKK1 via targeting miR‑103a‑3p. *Int. J. Mol. Med.* 46(3)**,** 1029-1038. doi: 10.3892/ijmm.2020.4672.

Liu, L., Zuo, Y., Xu, Y., Zhang, Z., Li, Y., and Pang, J. (2019). MiR-613 inhibits proliferation and invasion and induces apoptosis of rheumatoid arthritis synovial fibroblasts by direct down-regulation of DKK1. *Cell Mol. Biol. Lett.* 24(1)**,** 1-14. doi: 10.1186/s11658-018-0130-0.

Liu, X., Xu, H., Kou, J., Wang, Q., Zheng, X., and Yu, T. (2016). MiR-9 promotes osteoblast differentiation of mesenchymal stem cells by inhibiting DKK1 gene expression. *Mol. Biol. Rep.* 43(9)**,** 939-946. doi: 10.1007/s11033-016-4030-y.

Lv, C., Li, F., Li, X., Tian, Y., Zhang, Y., Sheng, X., et al. (2017). MiR-31 promotes mammary stem cell expansion and breast tumorigenesis by suppressing Wnt signaling antagonists. *Nat. Commun.* 8(1)**,** 1036. doi: 10.1038/s41467-017-01059-5.

Min, K., and Lee, S. K. (2019). EBV miR-BART10-3p Promotes Cell Proliferation and Migration by Targeting DKK1. *Int. J. Biol. Sci.* 15(3)**,** 657-667. doi: 10.7150/ijbs.30099.

Song, Q., Liu, H., Li, C., and Liang, H. (2021). miR-33a-5p inhibits the progression of esophageal cancer through the DKK1-mediated Wnt/β-catenin pathway. *Aging (Albany NY)* 13(16)**,** 20481. doi: 10.18632/aging.203430.

Tang, X., Lin, J., Wang, G., and Lu, J. (2017). MicroRNA-433-3p promotes osteoblast differentiation through targeting DKK1 expression. *PLoS One* 12(6)**,** e0179860. doi: 10.1371/journal.pone.0179860.

Wang, C., Liao, H., Sun, H., Zhang, Y., and Cao, Z. (2018a). MicroRNA-3064-3p regulates the differentiation of cementoblasts through targeting DKK1. *J. Periodont. Res.* 53(5)**,** 705-713. doi: 10.1111/jre.12554.

Wang, F., Hu, X., Cao, C., Zhao, Y., and He, S. (2018b). MiR-488 promotes fracture healing by targeting DKK1. *Eur. Rev. Med. Pharmacol. Sci.* 22(24)**,** 8965-8972. doi: 10.26355/eurrev_201812_16667.

Wang, W., He, Y., Rui, J., and Xu, M. Q. (2019). miR-410 acts as an oncogene in colorectal cancer cells by targeting dickkopf-related protein 1 via the Wnt/beta-catenin signaling pathway. *Oncol. Lett.* 17(1)**,** 807-814. doi: 10.3892/ol.2018.9710.

Wang, Z., Wang, J., Chen, Z., Wang, K., and Shi, L. (2018c). MicroRNA-1-3p inhibits the proliferation and migration of oral squamous cell carcinoma cells by targeting DKK1. *Biochem. Cell Biol.* 96(3)**,** 355-364. doi: 10.1139/bcb-2017-0015.

Weng, J., Zhang, H., Wang, C., Liang, J., Chen, G., Li, W., et al. (2017). miR-373-3p Targets DKK1 to Promote EMT-Induced Metastasis via the Wnt/-Catenin Pathway in Tongue Squamous Cell Carcinoma. *BioMed Res. Int.* 2017. doi: 10.1155/2017/6010926.

Wu, Z., Zhang, Y., Yang, Z., Zhu, Y., Xie, Y., Zhou, F., et al. (2021). Elevation of miR-302b prevents multiple myeloma cell growth and bone destruction by blocking DKK1 secretion. *Cancer Cell Int.* 21(1)**,** 1-13. doi: 10.1186/s12935-021-01887-y.

Xia, Z., Wang, Y., Sun, Q., and Du, X. (2018). MiR-203 is involved in osteoporosis by regulating DKK1 and inhibiting osteogenic differentiation of MSCs. *Eur. Rev. Med. Pharmacol. Sci.* 22(16)**,** 5098-5105. doi: 10.26355/eurrev_201808_15703.

Xiang, J., Fu, H. Q., Xu, Z., Fan, W. J., Liu, F., and Chen, B. (2020). lncRNA SNHG1 attenuates osteogenic differentiation via the miR101/DKK1 axis in bone marrow mesenchymal stem cells. *Mol. Med. Rep.* 22(5)**,** 3715-3722. doi: 10.3892/mmr.2020.11489.

Zeng, H. B., Dong, L. Q., Xu, C., Zhao, X. H., and Wu, L. G. (2020). Artesunate promotes osteoblast differentiation through miR-34a/DKK1 axis. *Acta Histochem.* 122(7)**,** 151601. doi: 10.1016/j.acthis.2020.151601.

Zhang, H., Yu, C., Chen, M., Li, Z., Tian, S., Jiang, J., et al. (2016). miR-522 contributes to cell proliferation of hepatocellular carcinoma by targeting DKK1 and SFRP2. *Tumor Biol.* 37(8)**,** 11321-11329. doi: 10.1007/s13277-016-4995-0.

Zhang, J., Tu, Q., Bonewald, L. F., He, X., Stein, G., Lian, J., et al. (2011). Effects of miR‐335‐5p in modulating osteogenic differentiation by specifically downregulating Wnt antagonist DKK1. *J. Bone Miner. Res.* 26(8)**,** 1953-1963. doi: 10.1002/jbmr.377.

Zhang, Z. C., Liu, J. X., Shao, Z. W., Pu, F. F., Wang, B. C., Wu, Q., et al. (2017). In vitro effect of microRNA-107 targeting Dkk-1 by regulation of Wnt/beta-catenin signaling pathway in osteosarcoma. *Medicine* 96(27)**,** 27. doi: 10.1097/MD.0000000000007245.

Zhao, X., Sun, S., Xu, J., Luo, Y., Xin, Y., and Wang, Y. (2018). MicroRNA-152 inhibits cell proliferation of osteosarcoma by directly targeting Wnt/β-catenin signaling pathway in a DKK1-dependent manner. *Oncol. Rep.* 40(2)**,** 767-774. doi: 10.3892/or.2018.6456.

Zhou, A., Diao, L., Xu, H., Xiao, Z., Li, J., Zhou, H., et al. (2012). β-Catenin/LEF1 transactivates the microRNA-371-373 cluster that modulates the Wnt/β-catenin-signaling pathway. *Oncogene* 31(24)**,** 2968-2978. doi: 10.1038/onc.2011.461.

Zhou, X., Wang, Y., Li, Q., Ma, D., Nie, A., and Shen, X. (2020). LncRNA Linc-PINT inhibits miR-523-3p to hamper retinoblastoma progression by upregulating Dickkopf-1 (DKK1). *Biochem. Biophys. Res. Commun.* 530(1)**,** 47-53. doi: 10.1016/j.bbrc.2020.06.120.

Zovoilis, A., Smorag, L., Pantazi, A., and Engel, W. (2009). Members of the miR-290 cluster modulate in vitro differentiation of mouse embryonic stem cells. *Differentiation* 78(2-3)**,** 69-78. doi: 10.1016/j.diff.2009.06.003.
